# Mechanical perturbations trigger endothelial nitric oxide synthase activity in human red blood cells

**DOI:** 10.1038/srep26935

**Published:** 2016-06-27

**Authors:** Shunmugan Nagarajan, Rajendran Kadarkarai Raj, Venkatesan Saravanakumar, Uma Maheswari Balaguru, Jyotirmaya Behera, Vinoth Kumar Rajendran, Yogarajan Shathya, B. Mohammed Jaffar Ali, Venil Sumantran, Suvro Chatterjee

**Affiliations:** 1 VascularBiology Laboratory, AU-KBC Research Centre, Chennai, Tamil Nadu, India; 2 Pondicherry university, Pondicherry, India; 3 Department of Biotechnology, Anna University, Chennai, Tamil Nadu, India

## Abstract

Nitric oxide (NO), a vascular signaling molecule, is primarily produced by endothelial NO synthase. Recently, a functional endothelial NO synthase (eNOS) was described in red blood cells (RBC). The RBC-eNOS contributes to the intravascular NO pool and regulates physiological functions. However the regulatory mechanisms and clinical implications of RBC-eNOS are unknown. The present study investigated regulation and functions of RBC-eNOS under mechanical stimulation. This study shows that mechanical stimuli perturb RBC membrane, which triggers a signaling cascade to activate the eNOS. Extracellular NO level, estimated by the 4-Amino-5-Methylamino-2′, 7′-Difluorofluorescein Diacetate probe, was significantly increased under mechanical stimuli. Immunostaining and western blot studies confirmed that the mechanical stimuli phosphorylate the serine 1177 moiety of RBC-eNOS, and activates the enzyme. The NO produced by activation of RBC-eNOS in vortexed RBCs promoted important endothelial functions such as migration and vascular sprouting. We also show that mechanical perturbation facilitates nitrosylation of RBC proteins via eNOS activation. The results of the study confirm that mechanical perturbations sensitize RBC-eNOS to produce NO, which ultimately defines physiological boundaries of RBC structure and functions. Therefore, we propose that mild physical perturbations before, after, or during storage can improve viability of RBCs in blood banks.

The work of Kosaka *et al*.^1^ suggested that hemolysate of erythrocyte membrane has endothelial nitric oxide synthase (eNOS) catalytic activity^1^. The study of Cortese-Krott *et al*.^2^ demonstrated that the red blood cells (RBCs) possess eNOS and produce nitric oxide (NO)^2^. The work of Kleinbongard *et al*.^3^ confirmed that RBC carry eNOS enzyme to generate NO production, and that eNOS localizes in the cytoplasm and plasma membrane of the RBC^3^. It is evident that shear stress can regulate NO production by RBC^4^,^5^. In addition, the modulation of NO levels due to activation or inhibition of RBC-eNOS can regulate deformability of RBC membranes^6^,^7^. However, the effects of routine physical perturbations on RBCs in motion have not been examined. This is of particular relevance, since RBCs have structural flexibility in order to fit themselves into both aorta and capillary loops as they traverse through the varied and versatile architecture of the vasculature. In the presence of such complex physical perturbations, RBC membranes can be altered transiently or for longer periods due to presence of a “shape memory”^8^. Also, localized strain at a given position on the biconcave RBC surface and resilience within the RBC membrane, may result in uneven physical perturbations which may transiently activate NO production^9^. RBCs also collide with each other and other cell types during their sojourn through blood vessels^10^. Therefore, our proposition is that colliding RBCs are always under “Off and On” mode of NO production in a given laminar flow condition. This occurs because the deformability of the RBC membrane alters as it undergoes transient changes in shape during each collision. In this context, we hypothesized that a tightly controlled mechano-transduction pathway regulates NO production in RBC. Hemo-rheological disturbances in the patho-physiological microenvironment are associated with aggregation and local accumulation of RBC in microvascular lumina, thus entailing disorders of blood flow. Abnormal blood flow and apparently static milieu of aggregated RBC in hematoma could potentially result in loss of a critical NO pool. Therefore, our primary experimental model involved measurement of NO production by static and mechanically perturbed human RBCs in suspension. After standardizing a reproducible mode of mechanical agitation for suspended RBCs, we measured relative and absolute levels of RBC derived nitric oxide (NO) by fluorimetric and ultrasensitive electrochemical methods, respectively. Since suspended RBCs were used, the upstream mechanism of NO production and RBC-eNOS signalling were investigated in detail. Importantly, we demonstrate that NO produced by suspended, mechanically perturbed, RBCs regulated important functions of endothelium. We also examined the possibilities of translating this knowledge into applications such as an improved strategy for blood storage.

## Results

### Mechanical perturbation promotes RBC-eNOS activity

**Figure 1:**
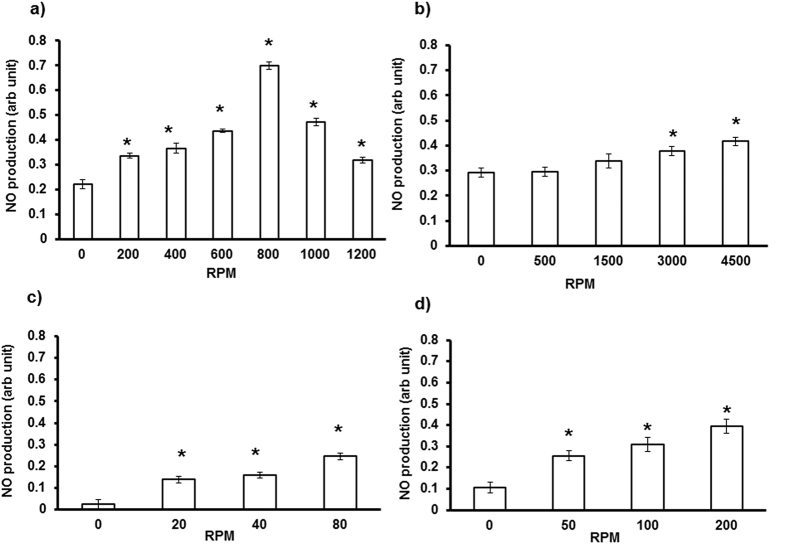
Physical perturbation induces RBC-NO production. RBCs were incubated with DAF-FM (5 μm) for 30 minutes, and subjected to different physical forces using centrifuge, shaker, rocker or vortex. A dose-dependent increase in NO production was observed in RBCs subjected to **(a)** vortex (200, 400, 600, 800 rpm), followed by decreased NO production at higher speeds (1000, 1200 rpm) (n = 3; *p < 0.001). Significant increase in NO production was observed in RBCs subjected to **(b)** centrifugation (3000, 4500 rpm) (n = 3; *p < 0.038), **(c)** rocker (20, 40, 80 rpm) (n = 3; *p < 0.001), and **(d)** shaker (50, 100, 200 rpm) (n = 3; *p < 0.004), versus control RBC (static).

Figure 1a shows a 3-fold increase in NO production in RBC subjected to vortex (200–800 rpm), followed by significantly decreased NO production at higher speed (1000–1200 rpm). Perturbation caused by centrifugation 4,500 rpm (Fig. 1b), rocker 80 rpm (Fig. 1c), and shaker 200 rpm (Fig. 1d) also caused significant increase in NO production versus control RBCs (0 rpm, static). Maximum NO production was observed in RBCs vortexed at 800 rpm for 20 seconds. Two assays were performed to determine if vortex conditions damaged RBC membranes. The first assay measured plasma free haemoglobin as a marker for RBC membrane integrity and morphology^11^. The second assay monitored levels of total ROS and peroxynitrite as markers of oxidative stress in vortexed RBCs with specific cell-permeant fluorescent probes^12^. We observed that vortex conditions did not significantly alter levels of free haemoglobin (Supplementary Fig. 1a); peroxynitrite or total ROS (Supplementary Fig. 1b). Hence, the above data clearly show that mechanical perturbation of RBC membranes during vortex conditions resulted in NO production without affecting integrity of the RBC membrane.

### Comparable activation of RBC-eNOS by mechanical perturbation and chemical agonists of eNOS

**Figure 2:**
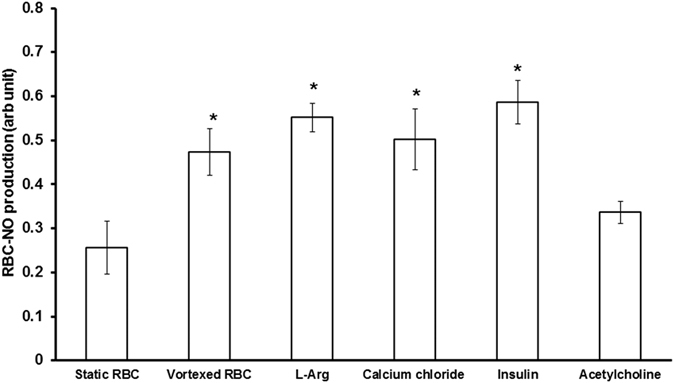
Chemical agonists and vortexing induce comparable levels of NO production by RBCs. RBCs were pre- incubated with insulin (5 μM), calcium chloride (1 mM) or L-arginine (1 mM) for 30 mins at 37 °C, followed by incubation with DAF-FM (5 μm) for 30 minutes prior to measurement of NO. RBCs treated with agonists showed a significant 2–3 fold increase in NO production versus untreated controls (*p < 0.042). A similar increase in NO was observed in vortexed RBC (800 rpm for 20 seconds) versus static controls (n = 3;*p < 0.001).

Physiological agonists of eNOS such as insulin, calcium chloride, and L-arginine; induced a significant 2–3 fold increase in NO production relative to static RBCs. Notably, the relative levels of NO produced by vortexed RBCs and RBCs treated with chemical agonists were similar (Fig. 2).

### Real time production of RBC-NO

**Figure 3:**
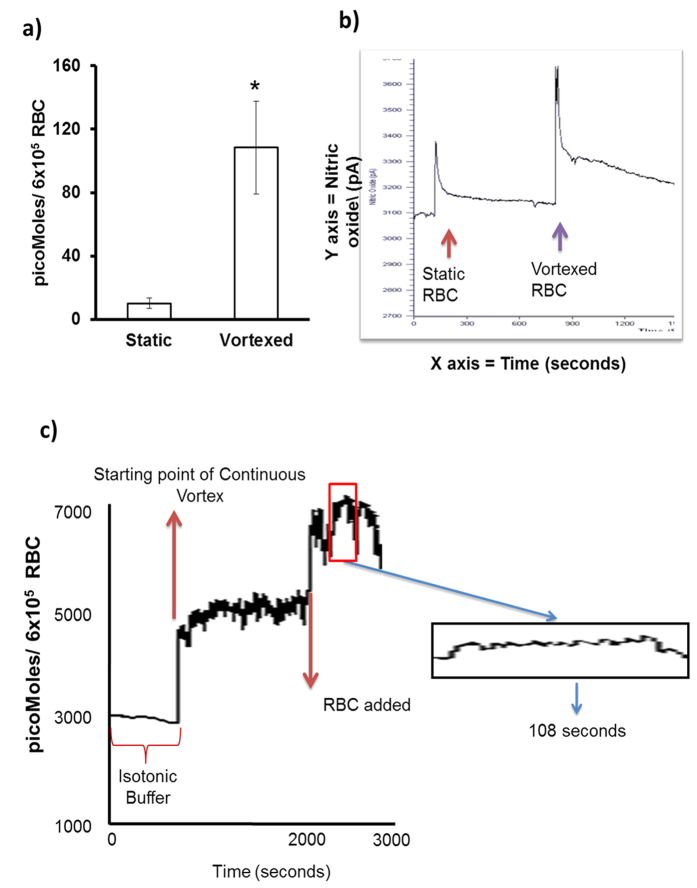
Measurement of NO in Vortexed RBC with Ultrasensitive NO electrode. Real time NO measurement in a suspension of RBCs (6 × 10^5^ RBC/mL) was carried out using an optically isolated, multi-channel free radical analyzer with an NO-selective membrane (Apollo 4000). **(a)** Vortexed RBC produced 108.415 pMoles of NO, whereas static RBC produced 10.19 pMoles of NO (n = 3; *p = 0.035, t-test). **(b)** Recording showing NO measurements from static and vortexed RBCs from same donor. **(c)** RBC-NO level recorded by continuous vortex mode. Real time NO measurement in a suspension of RBCs (1 × 10^6^ RBC/mL) was carried out using the NO-selective membrane. After stabilization with buffer, the instrument was subjected to sustained vortex in buffer (800 rpm) until it re-stabilized. At 2000 seconds, RBCs were added and vortexed, and the instrument re-stabilized for an additional 600 seconds. Vortexed RBCs continuously produced NO from 600 to 708 seconds (108 sec). In the first 20 secs, 5.98 μM NO were produced. A total of 6.07 μM NO were produced from 600 to 708 seconds (108 sec), (n = 3).

We monitored real time NO production by RBC in static and vortex conditions by an ultra-sensitive NO electrode^13^. Figure 3a,b shows vortexed RBC produced 10 fold higher levels of NO than static RBC. Thus, 6 × 10^5^ vortexed RBC produced 108.41 pM of NO after 20 seconds of vortexing, whereas static RBC produced 10.19 pM of NO. On a per cell basis, a single vortexed RBC produced 0.18 pM of NO, whereas a single static RBC produced 0.02 pM of NO. Figure 3c shows that continuously vortexed RBCs (1 × 10^6^) produced 6.07 μM (6.07 ± 0.03) of NO over a period of 108 seconds. Notably, a significant 98% of this NO (5.98/6.07 = 0.98) was produced during the first 20 seconds of vortex. These results are consistent with our earlier observation showing a maximum NO production in RBCs vortexed at 800 rpm for 20 seconds.

### Regulation of RBC-NO through membrane deformation

**Figure 4:**
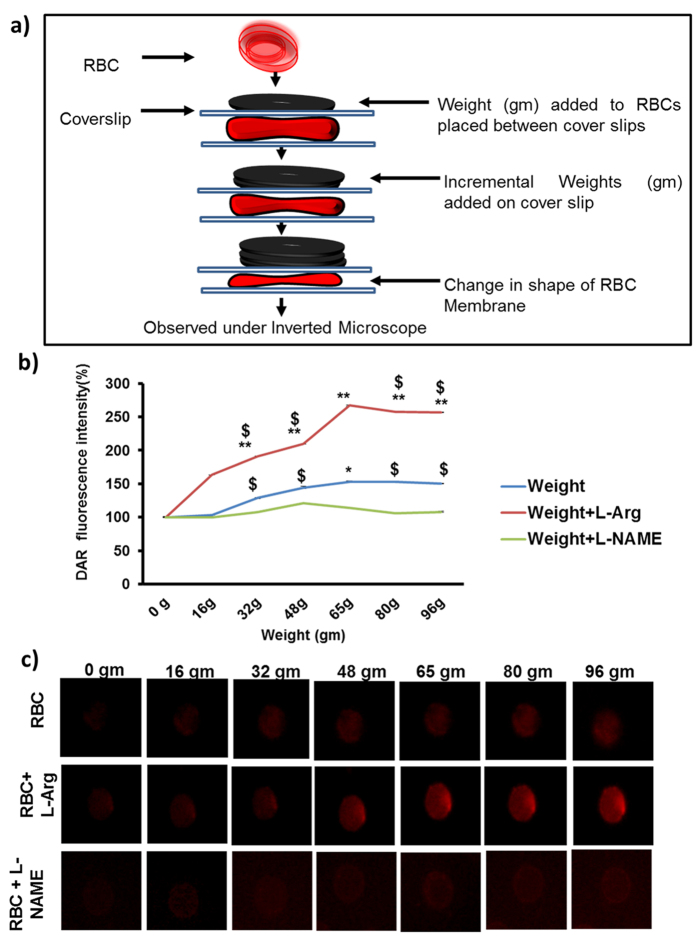
Physical pressure on RBC membrane induces NO production. **(a)** Schematic representation of physical perturbation of RBC. RBCs were pre-incubated with DAR in presence of NOS substrate (L-arginine) and NOS inhibitor (L-NAME) for 30 minutes at 37 °C. RBCs were placed between poly L-lysine coated cover slips, and placed under an inverted microscope. External weights (16 gm) were incrementally stacked on the coverslip at one minute intervals, and imaged after each interval. **(b)** Real time NO production was captured by fluorescence microscopy. When compared to 0 gram control, RBCs loaded with 65.15 g showed a significant increase in NO production (n = 3; *p = 0.000313). In presence of L-Arginine, RBCs loaded with all weights (apart from 16.38 gm), showed increased NO production when compared with 0 gram (control) (n = 3; **p = 0.00248; **p = 0.00035, **p = 3.77E-07, **p = 1.29E-06, **p = 0.000749). NO production by loaded RBCs in presence of L-Arginine was also greater than that observed in loaded RBCs without L-Arginine (n = 3; ^$^*p* = 0.000185, ^$^*p* = 0.0000186, ^$^*p* = 0.00238, ^$^*p* = 1.49E-06). Addition of L-NAME also decreased NO production by loaded RBCs. **(c)** Fluorescence images of RBC under mechanical perturbation were imaged by OlympusIX71 microscope using 60× magnification and oil-immersion objective lenses with a numerical aperture (NA) of 1.42.

In order to investigate the role of RBC membrane deformation in NO production, RBCs sandwiched between two cover slips were incrementally loaded with defined weights each (16 gm), and real time intracellular RBC-NO production was tracked by DAR imaging^14^. Figure 4a shows a schematic representation of experimental setup. Figure 4b shows that adding 64 gm weight caused a gradual and significant 1.50 fold increase in NO production when compared with controls without weight (0 gm). NO production by loaded RBCs in presence of L-Arginine was also greater than that observed in loaded RBCs without L-Arginine (n = 3; ^$^p = 0.000185, ^$^p = 0.0000186, ^$^p = 0.00238, ^$^p = 1.49E-06). Results showed that the NO produced by loaded RBCs was eNOS dependent, since presence of (L-arginine) enhanced NO production by loaded RBCs by 2 fold. Conversely, presence of (L-NAME) abrogated this increase in NO production. Figure 4c shows representative fluorescence images of RBCs after addition of weights at 1 min intervals. Further, we explored the role of membrane fluidity on NO production in vortexed RBC through fluorescence anisotropy studies. Increasing speeds of vortex resulted in a significant increase in fluidity of RBC membranes, which could in part be reversed by the NOS inhibitor L-NAME (Supplementary Fig. 2).

### Mechanical perturbation activates upstream signalling which induce RBC-eNOS

**Figure 5:**
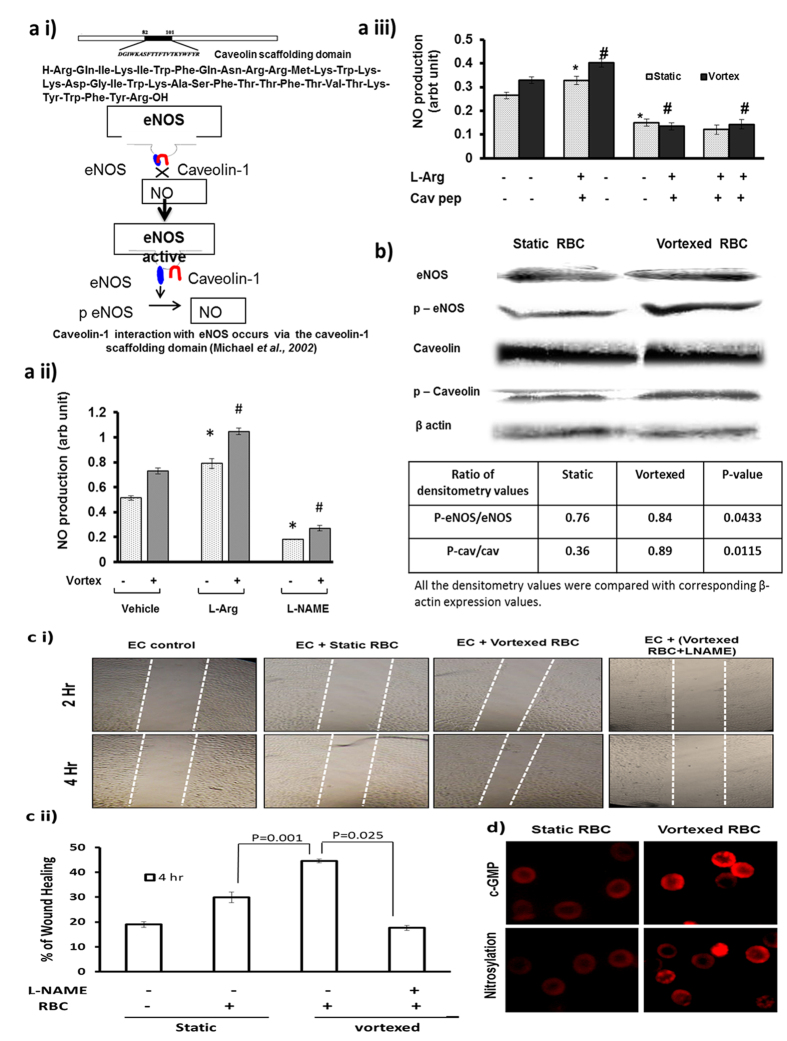
Production of NO by vortexed RBCs stimulates wound healing and is e-NOS dependent. **(a i)** Representative image shows role of caveolin in eNOS activation. Static or vortexed RBCs were incubated with e-NOS substrate (L-Arginine), NOS inhibitor (L-NAME) or caveolin scaffold domain (82–101) inhibitor for 30 minutes at 37 °C, and NO level was measured using DAF-FM. **(a ii)** Bar graph shows NO production was significantly increased in static RBCs in presence of L-arginine (n = 3; *p = 0.007), and decreased in presence of L-NAME (n = 3; *p < 0.001). Similarly, **(a iii)** NO production was significantly increased in vortexed RBCs in presence of L-arginine (n = 3; **p < 0.001) and decreased in presence caveolin scaffolding peptide (n = 3; ^#^*p* < 0.001). (**b)** Vortexing induced phosphorylation of RBC eNOS and caveolin-1. Static and vortexed RBC showed similar basal levels of eNOS and caveolin proteins in RBC ghosts. Vortexed RBCs showed increased levels of phosphorylated e-NOS (serine-1177) (p = 0.0433) and phosphorylated caveolin (Tyr-14) (p = 0.0115) compared with static RBC. **(c)** NO produced by vortexed RBC promotes wound healing in EAhy926 cells. Static and vortexed RBCs (3 × 10^6^/3 mL) were incubated with the wound created in EAhy926 monolayer for 30 minutes in a CO_2_ incubator at 37 °C. Distances migrated by wounded cells were imaged and measured at the 4h time point. (**c i)** Representative image at 0th and 4th hour was captured by IX71 Olympus fluorescence microscope using a 4X objective lens and NA 0.10. (**c ii)** Wounded cells treated with vortexed RBC showed 20% greater migration than wounded cells treated with static RBC (n = 3; *p = 0.001). **(d)** Immunofluorescence images of vortexed and static RBC show TRITC-positive spots stained by anti-cGMP and anti nitrocysteine antibodies. Images shows significant increased intensity of cGMP (n = 3; *^#^p* = 0.010; t-test) and nitrosylation (n = 3; *p < 0.001; t-test) in vortexed versus static RBC. Images were by OlympusIX71 microscope, 60× magnification and oil-immersion objective lenses with a numerical aperture (NA) of 1.42.

(Figs 1, 2, 3) demonstrated that different modes of mechanical perturbation of suspended RBC resulted in significant levels of NO production. It was therefore important to determine whether this NO was specifically produced by activation of RBC-eNOS. Figure 5a i) schematic representation image shows the unbinding of caveolin scaffolding domain from eNOS and eNOS activation. Figure 5a ii) shows that NO produced by static and vortexed RBCs is eNOS dependent since it was significantly stimulated by (L-arginine) and inhibited by (L-NAME). Caveolin-1 scaffolding domain peptide is a specific eNOS interacting protein, which negatively modulates eNOS activity^15^. Figure 5a iii) shows that interference of the eNOS-caveolin interaction with a caveolin-1 scaffolding domain peptide significantly attenuated NO production by RBC in static and vortex condition. These data proved that NO production by RBCs subjected to mechanical perturbation is catalyzed by eNOS. However the phosphorylation of eNOS at ser-1177 plays a critical role in its activation. Therefore, we analyzed expression of RBC-eNOS, caveolin-1, and their corresponding phosphorylation status by western blot. Both static and vortexed RBC expressed similar levels of eNOS and caveolin proteins in RBC ghosts. However, only vortexed RBC had increased levels of phosphorylated e-NOS (serine-1177) and phosphorylated caveolin (Tyr-14) when compared with static RBC (Fig. 5b). The data in Fig. 5b) was analyzed by densitometry, which confirmed that vortexed RBC showed a statistically significant increase in phosphorylated e-NOS and phosphorylated caveolin relative to static RBC.

### NO produced by mechanically perturbed RBC-eNOS regulates endothelial functions

We hypothesized that the NO produced by mechanically perturbed RBC-eNOS could regulate endothelial functions like cell migration, regulation of secondary signalling, and vascular sprouting. With respect to cell migration, a scrape wound-healing assay was performed on immortalized EAhy926 cells incubated with equal numbers of static or vortexed RBCs. Figure 5c(i and ii) showed that NO produced by vortexed RBCs induced a significant 20% increase in migration of wounded EAhy926 cells when compared with NO produced by static RBC. With respect to secondary signalling, immunofluorescence showed that NO derived from vortexed RBC could modulate levels of cGMP and nitrosylated RBC proteins (5d). In addition, the role of RBC derived NO in angiogenesis was confirmed by treating extra-embryonic vascular beds with vortexed RBC for 4 hours. Real time tracking demonstrated that NO from vortexed RBC significantly increased the number, length, size, and junctions formed in the vascular bed (Supplementary Fig. 4a,b). Thus, the NO produced by activation of RBC-eNOS in suspended, vortexed RBCs stimulated the important endothelial processes of cell migration, nitrosylation of RBC proteins and vascular sprouting.

### Band3 mediated changes in intracellular chloride levels lead to RBC-eNOS activation

**Figure 6:**
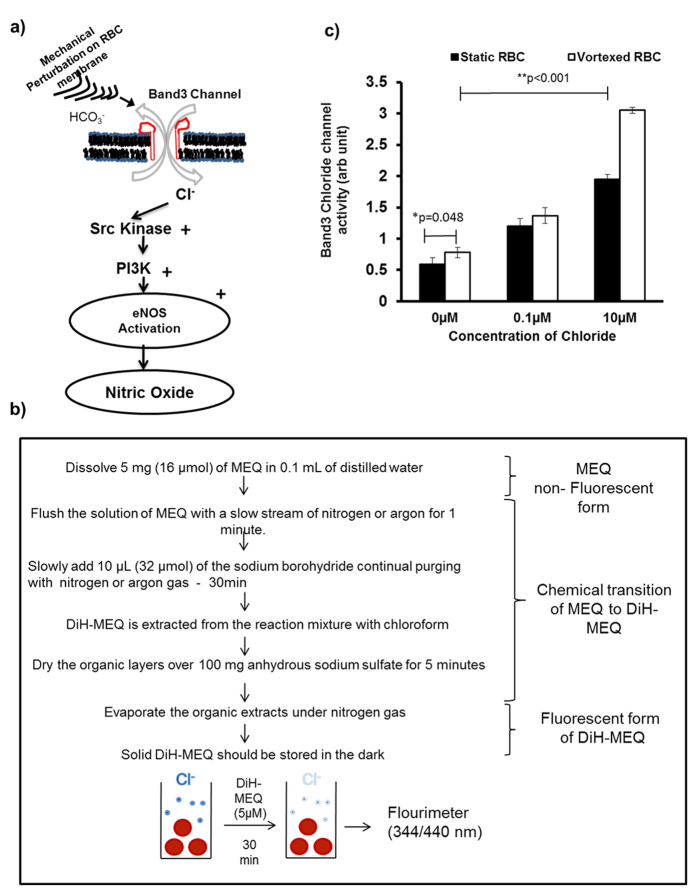
Band3-activated chloride influx in vortexed and static RBCs. **(a)** Schematic representation of NOS activation pathway in vortexed RBCs: Band3 chloride channel regulated by vortex mode mediates chloride influx which activates Src-Kinase/PI3K pathway for RBC-eNOS activation. **(b)** Flow diagram for synthesis of fluorescent form of DiH-MEQ from its non-fluorescent form. **(c)** RBCs were incubated with chloride free ringer solution and DiH-MEQ for 45 minutes. Chloride influx was higher in vortexed versus static RBCs at 0 μM calcium chloride (n = 3; *p = 0.048). Incubation with increasing concentrations of exogenous calcium chloride caused a dose dependent increase in chloride influx into vortexed and static RBCs versus untreated RBCs (n = 3; **p < 0.001; ***p < 0.001).

**Figure 7:**
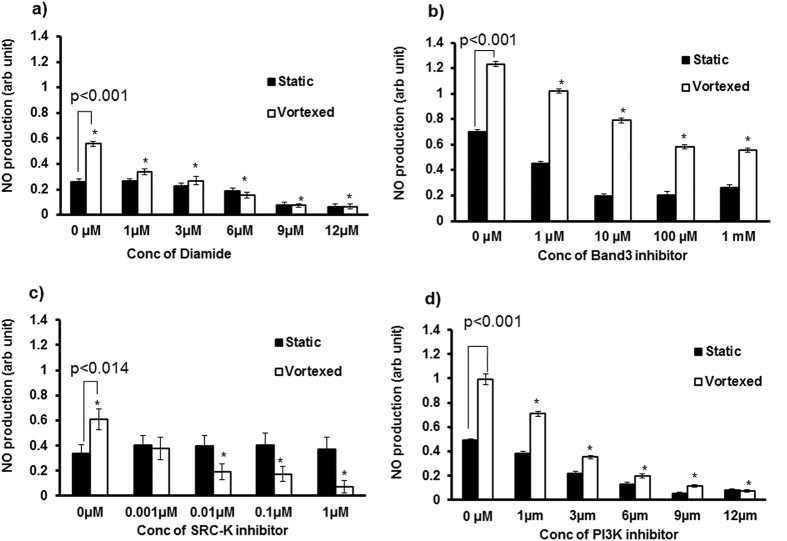
Inhibitors of NOS activation decrease NO production by static and vortexed RBCs. RBCs were pre-incubated with diamide or specific inhibitors of band3, Src kinase, or PIK3. Washed RBCs were incubated with DAF-FM (5 μM) for 30 minutes, and subjected to static or vortex mode, followed by measurement of NO production at 495/515 nm. Vortexed RBCs produced significantly more NO than static RBCs in the absence of inhibitors. Increasing concentrations of diamide **(a)**, band3 inhibitor **(b)**, src-kinase inhibitor **(c)**, and PIK3 inhibitor **(d)**; caused significant and dose-dependent decrease in NO production by vortexed RBCs compared with static RBCs (n = 3; *p ≤ 0.014).

Vortexed RBC specifically activates RBC-eNOS. Therefore, it was necessary to determine if molecules required for upstream eNOS signalling were also activated during vortex conditions in suspended RBCs. Previous studies have shown that influx of chloride ion through the band3 ion channel is an early step in activation of RBC-eNOS (Fig. 6a). In order to demonstrate this crucial first step in upstream of eNOS signalling, we synthesized a membrane permeable, chloride sensitive probe 6-Methoxy-N-ethylquinolinium Iodide (MEQ)^16^, and measured influx of chloride in vortexed RBCs versus static controls. (Figure 6b) shows the protocol used to chemically convert MEQ into its membrane permeable form, Di-HMEQ. Fluorescence intensity of Di-HMEQ at 344/440 nm is inversely proportional to chloride influx into RBC. Figure 6c demonstrates that chloride influx was significantly higher in vortexed versus static RBCs at 0 μM Calcium chloride (Cacl_2_) concentrations. Vortexed RBCs incubated with 10 μM CaCl_2_ also showed a statistically significant 2.50–3.0 fold increase in chloride influx when compared with static RBCs. These data strongly suggest that band3 protein mediates chloride transport across the RBC membrane during mechanical perturbation caused by vortexing. In order to examine the role of other molecules in upstream eNOS signalling, vortexed RBCs were treated with diamide, an SH-oxidizing agent which disrupts protein-lipid interactions, or specific inhibitors of band 3, Src Kinase, and PI3-Kinase. Using these inhibitors, we proved that intact protein-lipid interactions in the membrane (Fig. 7a), and activation of Band3 (Fig. 7b), Src kinase (Fig. 7c), and PI3K proteins (Fig. 7d); were responsible for RBC-eNOS activation under vortex conditions. These data suggest that vortex conditions can alter membranes of suspended RBCs, and activate the band 3 anion exchanger. The subsequent influx of chloride activates the Src kinase/PI3K pathway leading to phosphorylation of eNOS (at ser1177), and eNOS activation.

### Mechanical perturbation improves SNO-Hb level in RBC proteins

**Figure 8:**
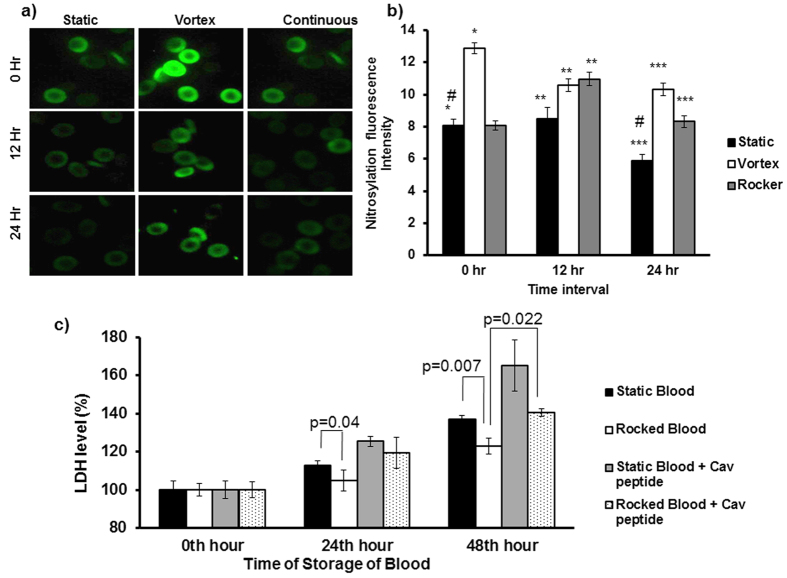
Mechanical perturbation improves nitrosylation levels and viability of RBCs in stored blood. Human blood from 3 healthy volunteers was collected and stored in static or continuous rocker condition (15 rpm) for 0–24 h at 4 °C. Other stored samples were vortexed (800 rpm for 20 seconds) during storage at 3 time points (0, 12 hr, and 24 hr). **(a)** Nitrosylation of RBCs in stored blood samples subjected to vortex or rocker conditions was investigated by immunostaining with anti-nitrocysteine antibody. RBCs were imaged by OlympusIX71 microscope, 60× magnification and oil-immersion objective lenses with a numerical aperture (NA) of 1.42. **(b)** Nitrocysteine levels gradually decreased in static RBC stored for 24 hours compared to 0 h (n = 3; ^#^*p* = 0.025). At 0 hours, RBC in vortexed blood showed higher nitrocysteine levels compared to static RBC (n = 3; *p < 0.001). After 12 hrs and 24 hrs of storage, RBC pre-conditioned by rocker or vortex treatment showed higher nitrocysteine levels than that observed in static blood stored for 12 hr and 24 hr (n = 3; **p < 0.03; and ***p < 0.004, respectively). **(c)** LDH activity in plasma at 0, 24 hr, 48 hr. LDH activity increased in static blood stored at 24 hr and 48 hr compared with rocked blood (n = 3; *p* = 0.04; *p* = 0.007). Inhibition of eNOS by caveolin peptide in stored blood subjected to rocker, decreased RBC viability and lead to increased plasma LDH when compared to similarly treated blood without caveolin peptide at 48 hr (n = 3; *p* = 0.022).

Re-nitrosylation of stored blood could improve oxygen delivery from RBCs into tissues during blood transfusion^17^. We examined whether vortex and rocker conditions could stimulate SNO-Hb of RBCs in stored blood. Immunofluorescence images (Fig. 8a,b) shows that vortex treatment caused a significant 1.60 fold increase in SNO-Hb levels of RBC in fresh blood. Vortexed RBC after 12^th^ and 24^th^ hour of storage also showed significantly higher levels of SNO-Hb than that of static blood at same time of storage respectively. Continuous rocking treatment of blood during 12^th^ and 24^th^ hour of storage also maintained higher levels of SNO-Hb of RBC. Notably, RBCs in vortexed blood showed significantly greater SNO-Hb in comparison to that of RBCs from blood in rocking condition at the 24^th^ time point. Since viability of RBC in stored blood is essential, we estimated level of plasma LDH as a biomarker for haemolysis. Plasma LDH level was significantly increased at 24^th^ and 48^th^ in static blood compared with rocked blood (Fig. 8c). Addition of caveolin peptide to static and rocked blood further increased plasma LDH levels. These data strongly suggest that caveolin peptide inhibited RBC-NO production and SNO-Hb of RBC proteins in vortexed condition. The decreased SNO-Hb of RBC proteins in stored blood resulted in decreased RBC viability and higher levels of LDH release.

## Discussion

In contrast to endothelium RBC enjoys higher order of freedom since it flows along with the blood circulation. Laminar shear stress activates eNOS by phosphorylating serine and tyrosine moieties in eNOS in endothelium^18^. RBC-eNOS activity and intracellular NO levels were increased in immobilized RBC exposed to well-defined fluid shear stress^19^. Under simple shear flow, only two motions, “tumbling” and “tank-treading,” have been described experimentally that relate to cell mechanics of the RBC^20^. Vascular network includes one-to-two branching (bifurcation) and one-to-many branching (trifurcation, quadfurcation and so on)^21^. Therefore, nature of fluidics, flow pattern, and shear have wide range of possibilities in biological vascular network. When RBC is placed in this heterogeneous pattern of fluidics, it experiences various kinds of flow associated events. Therefore, it is very difficult to simulate the *in-vivo* conditions, which RBCs experience in vascular milieu. We deem that physical perturbation we have used would closely represent turbulence and disturbed flow situations *in vivo* and its effects on RBC. The results suggest that RBC deformation in constricted vessels may increase NO levels in the RBC, and favor vasodilation, thereby providing an important role for RBC in regulating the circulation. Apart from “flow” factors RBC are colliding with each other, with other cell types and with the inner surface of vascular lumen in a routine fashion. Our proposition is that colliding RBC are always under “Off and On” mode of NO production in a given laminar flow condition because the RBC change their shape transiently each time one RBC collides with another cell or endothelium. First, we compared different modes of physical perturbation and found that mechanically vortexed RBC in suspension reproducibly produced higher levels of NO than static RBC. Interestingly, we observed that micromolar levels of NO production were sustained in the vortexed RBC for upto 108 seconds. Direct RBC trapping and manipulation have been reported in the literature^22^. Using optical tweezers, we could demonstrate that increased DAR fluorescence was observed in a single trapped RBC but not in a free RBC (Supplementary Fig. 3a,b). This experiment further proved that single RBC subjected to a measurable force undergoes deformation which leads to production of detectable levels of NO. We then blocked eNOS activity in the RBC by incubating the RBC with caveolin-1 scaffolding domain peptide which is a specific inhibitor of eNOS activity. This eNOS specific approach confirmed that physical perturbation activates eNOS in the RBC to produce NO. The results confirmed that deformity of RBC membrane leads to the production of NO from eNOS. It is a known fact that NO reacts in a nearly diffusion-limited reaction with oxyhemoglobin and deoxyhemoglobin to form methemoglobin and iron-nitrosyl-hemoglobin. However, the NO scavenging property of free Hb is very different from that of bound sub-cellular Hb of RBC. In particular, the NO scavenger and vasopressor effects of hemoglobin present in RBC are limited by compartmentalization of hemoglobin within the erythrocyte. Therefore, we propose that the RBC membrane has unique sub-membrane properties that limit the rate of NO-hemoglobin reactions by approximately 600-fold^23^,^24^,^25^. This attenuated interaction between NO-hemoglobin would permit NO release which is then detected by our assays on static and vortexed RBC’s. We suggest that vortexed RBCs are transiently subjected to an increase in NO-hemoglobin interactions. This would explain the increased NO produced in vortexed RBCs versus static controls (Figs 1, 2, 3).

At this juncture we ask the question “How the physical perturbation of RBC translate into the activation of eNOS and NO production?” To address this question we compared the RBC preparedness for responding to membrane perturbations in suspension with dedicated NO producing endothelial cells in suspension, and observed that RBC is more sensitive in responding to physical perturbations and producing NO than endothelial cells (data not shown).

Our results conceptualized that mechanical perturbations alters the order of freedom in the RBC membrane, which further invokes Band3 –src kinase – PI3K activation and converges on eNOS phosphorylation. The released NO from RBC will have 3 immediate targets 1) The RBC itself an autocrine loop, 2) Other RBCs and blood cells in vicinity and 3) Vascular inner lumen the endothelium. We performed two cell based assays to understand the role of agitation based RBC derived NO on RBC membrane and endothelium. Results of the experiments confirmed that RBC-NO produced by physical perturbations is functionally active for both autocrine and remote targets. Hemorheological disturbances in the patho-physiological microenvironment are associated with intensified RBC aggregation and the subsequent local accumulation of RBCs in the microvascular lumina can entail disorders of the blood flow. Our results show that vortexed RBCs can significantly increase chick embryo angiogenesis and wound healing when compared with static RBCs (Supplementary Fig. 3 and Fig. 5c i,ii). These observations clearly indicate that agitation associated NO production by RBC has functional implications. Present work transpired that mild physical perturbations stimulate NO production machinery in the RBC. We envisage that exercise enforces RBC to traverse through 2 micron capillary ends of the vascular tree with a faster rate that significantly compromises the shape and physical dimensions of RBC. When RBC was exposed to shear stress by filtration through 5 micron diameter pores under 10 cm H_2_O pressure, generating a wall shear stress of approximately 110 Pa^5^.

RBCs are known to form parachute shapes under flow in microvessels or glass capillaries with diameters between 3 and 13 μm^26^,^27^,^28^,^29^,^30^. Evidence from high-speed cinephotography of the microcirculation in the mesentery of the dog shows that the shape of the red blood cell is changed during its flow through capillaries from a biconcave disk to a paraboloid with a hollow bell-like^31^. The work of Filipovic *et al*.^32^ indicates that electro stimulation of the whole body (WB-EMS)^32^ represents a useful and time-saving addition to conventional training sessions to improve RBC deformability and possibly oxygen supply to the working tissue and thus promoting general force components in high performance sport. Although our study (Fig. 4b) did not simulate capillary-RBC interactions, our experimental models of vortexed RBCs and RBCs subjected to increasing weights, represent in part, the tremendous shape compromising properties of RBCs which sojourn through varied architecture and diameter of blood vessels. Our data on NO release in these experimental models has implications on the *in vivo* conditions under which NO production by RBC occurs. Based on the previous literature and our observations, we propose that enhanced rate of blood circulation increases the frequency of collision of RBC with RBC and other blood cells and vascular walls, and thereby NO production.

A recent study showed that storage of blood leads to significant inhibition of eNOS activity in the RBCs^33^. To explore the anticipated applications of the knowledge derived from the present study we developed a preliminary experimental strategy to enhance NO production by physical perturbation of RBC to improve the post thaw parameters of stored blood since approximately 85 million units of blood are administered worldwide each year, and therefore, transfusion-related morbidity and mortality is a major public health concern worldwide. RBC-NOS regulates NO production which can then cause S-nitrosylation of RBC hemoglobin^34^. A recent work suggested that re-nitrosylation could be a better strategy to store blood in the blood banks^17^. The study confirmed that banked blood shows reduced levels of s-nitrosohemoglobin (SNO-Hb), which in turn impairs the ability of stored RBC to affect hypoxic vasodilation. The result of the work indicates a solution, which prescribes nitrosylating the RBC before storing them in the bank.

Our work specifically offers a least invasive way to tackle the problem. We confirmed that mechanical perturbation facilitates nitrosylation of RBC proteins via eNOS derived NO under the perturbed conditions (Fig. 8). Therefore, we suggest that coupling mild physical perturbations like rocking and shaking of stored blood before, after or during the storage steps of blood banking protocol. Performing limited experiments using routine blood storage protocols we have shown that nitorsylation via physical perturbations of RBC could be a fruitful option for a better storage of the blood in the bank. If these data are replicated in clinical conditions, re-nitrosylation therapy by least invasive and controlled physical disturbances of RBC could have significant therapeutic implications in the care of millions of patients worldwide.

### Materials and methods

Dulbecco’s modified Eagle’s medium (DMEM) was purchased from PAN-Biotech. Fetal bovine serum was from Invitrogen Life technologies, EAhy926 cell lines. 4-Amino-5-Methylamino-2′, 7′-Difluorofluorescein Diacetate (DAF-FM) was purchased from Invitrogen, NY, USA. Diaminorhodamine-4M-Acetoxymethylester (DAR-4M-AM), eosin-5-maleimide (EM), diamide, 4-(4′-Phenoxyanilino)-6, 7-dimethoxyquinazoline and citrite-phosphate-dextrose solution with adenine (CPDA), Drabkin’s reagent were purchased from Sigma Chemical. 6-Methoxy-N-ethylquinolinium Iodide (MEQ), 2′, 7′-dichloro-fluorescin diacetate (DCFH-DA) and dihydrorhodamine-123 (DHR-123) were purchased from Molecular probes. Caveolin-1 scaffolding domain peptide (C1-SD_82–101_) was purchased from Cal Biochem. Anti-eNOS (ser-1177), Anti p-eNOS (ser-1177) and Wortmanin purchased were from Santa Cruz Biotechnology. Anti caveolin-1, anti p-caveolin-1, anti cGMP, anti nitrocysteine were purchased from Abcam. Other chemicals were of laboratory grade and obtained commercially.

### Blood sampling and isolation of RBC

The protocols used in this study were approved by the Institutional Bio-safety and Ethical Committees (IBEC) of AU-KBC and the methods were carried out in accordance with the approved guidelines. Informed consent was collected from each healthy participant according to the protocol approved by IBEC of AU-KBC. Blood samples were collected from Volunteer Health Service and Lion’s blood bank, Chennai, India. Venous blood was taken from the antecubital vein. Red Blood cells (RBCs) were separated from leukocytes and platelets by centrifugation (800 × g, 4 °C, 10 min). RBC pellet was washed thrice and re-suspended in isotonic solution to a hematrocrit of 40% for all experiments. All the experiments were done using freshly prepared RBC suspensions and equal level of RBC (1 × 10^6^).

### Physical perturbation induces RBC-NO production

Suspended RBCs were subjected to mechanical perturbation by 4 different instruments (vortexer, centrifuge, shaker and rocker). Isolated RBC was subjected to centrifugation (0–4500 rpm) for 1 min. RBC was vortexed for 20 seconds at various speeds; the corresponding rpm (0–1200 rpm) was measured using a Tachometer. RBCs were also perturbed in an orbital shaker (0–200 rpm), or with a rocker at (0–80 rpm) for 1 min. NO production was measured using Varian Cary Eclipse UV–Vis Fluorescence spectrophotometer by DAF-FM at 495/515 nm.

### Comparative study of agonists and mechanical force on RBC-NO production

RBC suspensions were treated with or without insulin (5 μM), acetylcholine (5 μM), calcium chloride (1 mM) and L-arginine (1 mM) for 30 minutes. RBC suspensions were vortexed (800 rpm for 20 seconds) and NO measured using Varian Cary Eclipse UV–Vis Fluorescence spectrophotometer by DAF-FM at 495/515 nm.

### Ultrasensitive NO electrode

NO measurement was carried out at 37 °C using Apollo 4000, an optically multi-channel free radical analyzer with an NO selective membrane^13^. The NO selective membrane was calibrated with isotonic buffer prior to experiments. Briefly, freshly isolated RBCs (6 × 10^5^/ml) were suspended in isotonic buffer and the equilibrated electrode was placed such that’s tip was 10 mm above the RBC suspension. A static RBC suspension was used to monitor NO levels, and the same sample was then vortexed for 20 seconds prior to measurement of NO production. The NO selective membrane was then calibrated for the continuous vortex mode (800 rpm). RBCs were added, and then subjected to continuous vortexing. The NO production by these vortexed RBCs was continuously measured for a period of 108 seconds.

### RBC membrane perturbation imaging by DAR-4M-AM

RBCs were added to poly l-lysine coated cover slips and incubated with DAR-4M-AM^14^ for 30 mins at 37 °C. It was placed in the inverted microscope and external physical force was exerted by sequentially stacking defined weights at 1 minute intervals. Real time NO production was captured by fluorescence microscopy (Olympus IX71) at 60X. Fluorescence intensity of red blood cells was calculated by AdobePhotoshop7.0.

### Endogenous inhibition of eNOS by caveolin-1 scaffolding domain

Static RBCs suspensions were incubated for 30 min at 37 °C with L-arginine (1 mM) or L-NAME (1 mM). Static and vortexed RBCs were also incubated with caveolin- 1 peptide^16^ (10 μM) for 30 minutes at 37 °C, prior to measurement of NO production was measured using Varian Cary Eclipse UV–Vis Fluorescence spectrophotometer by DAF-FM at 495/515 nm.

### Western blot

RBC ghost membranes were prepared by osmotic lysis^35^. RBC ghosts were lysed in lysis buffer 50 mM Tris-HCl (pH 8.0), 0.1 mM EGTA, 0.1 mM EDTA, 1% TritonX-100, 1% SDS for 10 min on ice. Protein concentrations were determined by Lowry method using bovine serum albumin as a standard^36^. Electrophoresis of lysed RBC ghosts was performed with 100 μg of protein per well on 10% gels (SDS-PAGE). Resolved proteins were transferred to nitrocellulose membranes, blocked with 5% BSA, and probed with the desired primary antibody (1:1000) overnight at 4 °C. Washed blots were incubated with appropriate secondary antibody (1:2500) (anti-rabbit IgG) conjugated to Horseradish Peroxidase for two hours and the protein bands were visualised by adding substrate H_2_O_2_/3,3′,5,5′-Tetramethylbenzidine.

### Wound healing assay

Wound healing assay was modified in accordance with the method of Siamwala *et al*.^37^ EA.hy926 cells were incubated with 1 × 10^6^ RBC/ml (from static or vortex condition) for 30 minutes. RBC’s were removed and the cells were incubated for 4 hours. Bright field images of 4× magnifications with inverted microscope were taken in order to measure rate of wound healing. Image J image software was used for quantification.

### Immunofluorescence (IF) studies

RBCs were fixed with 0.25% glutaraldehyde^38^ in PBS for 30 minutes and immunolabelled with primary polyclonal antibodies against eNOS, eNOS-Ser1177, cav-1, cav-1Tyr19, cGMP and S-Nitrosohemoglobin (SNO-Hb) (1:1000); in PBS with 1% BSA overnight at 4 °C. Washed RBCs were then incubated with secondary goat anti-rabbit antibody conjugated with Fluorescein isothiocynanate (FITC) or Tetramethylrhodamine (TRITC) at a dilution of 1:2500 for 1 hour and observed with a fluorescent microscope (Olympus IX71) at 60X. Fluorescence intensity was calculated with an image analysis module of Adobe Photoshop ver.7.0.

### Measurement of intracellular chloride (Cl^−^)

Intracellular (Cl^−^) was measured by the Cl^−^ sensitive fluoroprobe^16^. RBCs from static and vortex conditions were incubated with diH-MEQ (50 μM) for 30 minutes in chloride free-buffered solution at 37 °C. The trapped dye in the supernatant was measured using Varian Cary Eclipse UV–Vis Fluorescence spectrophotometer at 344/440 nm. Since the DiH-MEQ fluorescence gets quenched in the presence of chloride ion extra-cellular fluorescence intensity of the DiH-MEQ has an inverse relationship with Band 3 chloride channel activity. The data presented as “Band 3 Chloride channel activity (arbitrary unit)”.

### Investigation of upstream eNOS signalling in static and vortexed RBCs

Static and vortexed RBC were incubated with inhibitors of different proteins known to activate eNOS. Membrane stabilizer diamide was used at (0 μM, 1 μM, 3 μM, 6 μM, 9 μM, 12 μM), Band3 anion exchanger channel was inhibited by EM^39^ (0 μM, 1 μM, 10 μM, 100 μM, 1 mM), the src-kinase-1^40^ was inhibited by 4-(4′-Phenoxyanilino)-6, 7-dimethoxyquinazoline, (0 μM, 0.001 μM, 0.01 μM, 0.1 μM, 1 μM), and phosphatidylinositol-3-kinase (PIK3) was inhibited by wortmanin^41^ (0 μM, 1 μM, 3 μM, 6 μM, 9 μM, 12 μM). DAF-FM was used to measure NO production by Varian Cary Eclipse UV–Vis Fluorescence spectrophotometer at 495/515 nm in static and vortexed RBC incubated for 20 mins at 37 °C.

### Effect of mechanical perturbation on SNO-Hb of RBCs in stored blood

Blood from healthy volunteers was incubated with CPDA solution at (1:9) ratio at 4 °C. SNO-Hb was visualized by (IF) using anti nitrocysteine antibody after storage of blood for 0 hr, 12 hr and 24 hr. During storage RBCs were separated from blood and vortexed at 800 rpm for 20 seconds before measuring SNO-Hb. Similarly, SNO-Hb level was measured after blood was placed on a rocker (15 rpm) for 0 hr, 12 hr or 24 hr at 4 °C.

### Effect of mechanical perturbation on LDH release by RBCs in stored blood

Blood (1 mL) was collected from 3 healthy donors. It was stored under static condition or on a continuous rocker (15 rpm) upto 48 hr at 4 °C. Plasma LDH level was measured by a kit (Agappe diagnostics Ltd)^42^.

### Statistical analysis

All experiments were performed in triplicate (n = 3) unless otherwise specified. Data were analysed with sigma stat. T-test was used when two conditions were compared and one-way Anova used for multiple comparisons. Values of p ≤ 0.05 were selected as showing a statistically significant difference.

## Additional Information

**How to cite this article**: Nagarajan, S. *et al*. Mechanical perturbations trigger endothelial nitric oxide synthase activity in human red blood cells. *Sci. Rep.* **6**, 26935; doi: 10.1038/srep26935 (2016).

## Supplementary Material

Supplementary Information
