# Supplementary material for: Mechanical perturbations trigger endothelial nitric oxide synthase activity in human red blood cells
Source: Sci Rep. 2016 Jun 27;6:26935. doi: 10.1038/srep26935 (PMC4921846; doi:10.1038/srep26935)
Supplement: Supplementary Information [file srep26935-s1.pdf]

Supplementary information

Mechanical perturbations triggers endothelial nitric oxide synthase activity in red blood cells

#Shunmugan Nagarajan[1], #Rajendran Kadarkarai Raj[1], Venkatesan Saravanakumar[1], Uma Maheswari Balaguru [1], Jyotirmaya Behera[1], Vinoth Kumar Rajendran[1], Yogarajan Shathya[1], B.Mohammed Jaffar Ali[3],Venil Sumantran[1] & Suvro Chatterjee[1,2,*]

***Corresponding author:** Dr. Suvro Chatterjee, E-mail address: soovro@yahoo.ca

Materials and methods

Estimation of plasma free hemoglobin level (fHB)

The fHB level in whole blood at static and subsequent vortex condition was measured in each sample through colorimetric assay using the Drabkin's reagent (Sigma-Aldrich, Saint Louis, Missouri, USA)[13].

Markers of oxidative stress in vortexed RBCs

To monitor levels of peroxynitrite by DHR-123 and total Reactive Oxygen species (ROS) by DCF-DA in RBC before and after vortexing, RBCs were incubated with DHR-123 (5µM) or DCF-DA (5µM) for 30 minutes[14] and washed with PBS. Fluorescence intensities of RBCs were captured by fluorescence microscopy (Olympus IX71) in real time, and were used to calculate levels of peroxynitrite and total ROS by an image analysis module of Adobe Photoshop ver.7.0.

Fluorescence Anisotropy Assay of RBC membrane. Effects of external physical force on membrane fluidity of RBCs were studied by fluorescence polarization. The sample containing the fluorescent probe 5-Iodoacetamidofluorescein (5-IAF) [41] was excited with linear polarized light and the vertical and horizontal components of the intensity of emitted light were measured. Polarization (P) is calculated using the following equations {Polarization (P) = Iv - Ih / Iv+ Ih where Iv is the intensity parallel to the excitation plane and Ih is the emission perpendicular to the excitation plane}. Anisotropy (rH) is directly related to polarization by the equation, {anisotropy (rH) = 2 x P/ 3- P} Anisotropy values are inversely related to membrane fluidity.

RBC in static or vortexed conditions were labelled by incubating cells with 5-IAF (0.04 mM) at 37˚C for 10 minutes in the dark. The washed cell suspension ($1x10^6$) was excited at 380 nm and emitted fluorescence was measured at 480 nm with a Cary Eclipse spectrofluorometer. I v and I h values were used to calculate polarization and anisotropy. RBC pre-treated with L-NAME was used as a negative control.

RBC deformation under optical trap

Single RBC deformation was studied by laser trap. RBCs were incubated with DAR for 30 mins and placed in optical tweezer apparatus built with Nikon Eclipse TE 2000-U microscope, Microscope Objective (NA – 1.4), Trapping laser (Infra red- 1064nm) and Fluorescence excitation source (Green laser – 532 nm) were used. Experiments were performed at room temperature[27].

Supplementary figures

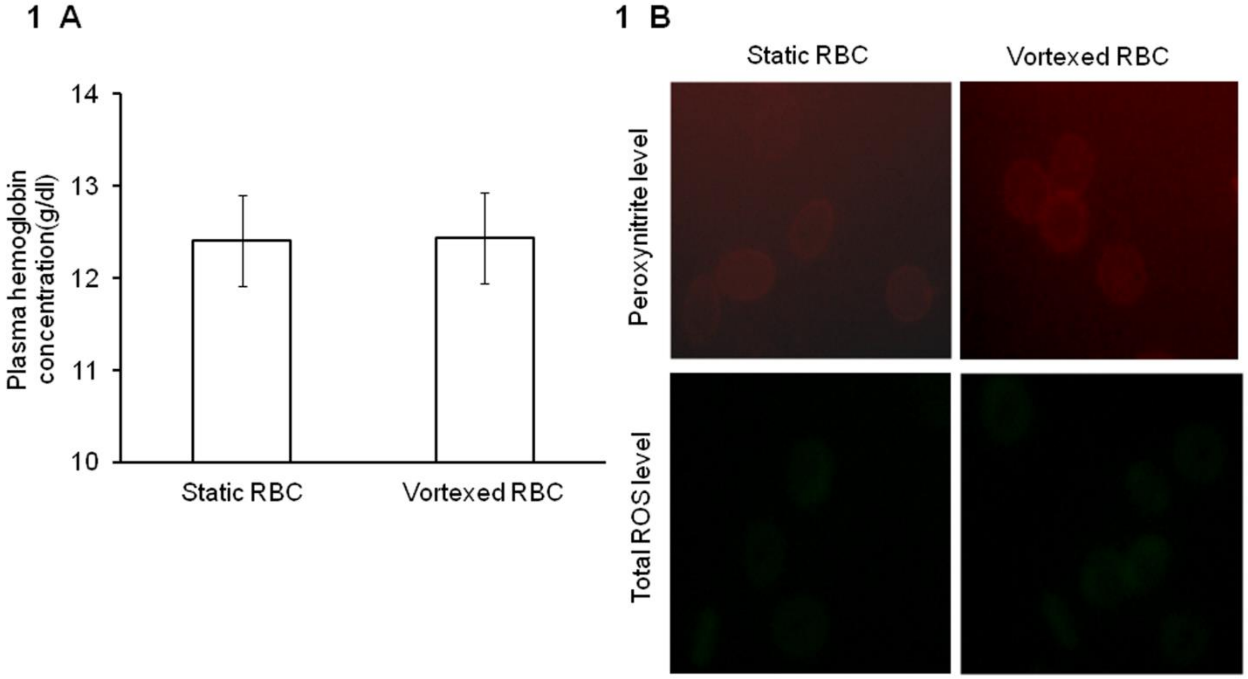

Supplementary Figure 1. Hemoglobin concentration in plasma of vortexed blood and monitoring oxidative stress. (A) Hemolysis of RBC was measured in plasma using Drabkin's Reagent. There was no significant difference in levels of hemolysis between static and vortexed RBCs (n = 3). **(B)** Markers of Oxidative stress in vortexed RBCs. RBC in static and vortexed conditions were incubated with DHR-123 (5μM) or DCF-DA (5μM) for monitoring levels of peroxynitrite and total ROS respectively (n = 3).

2

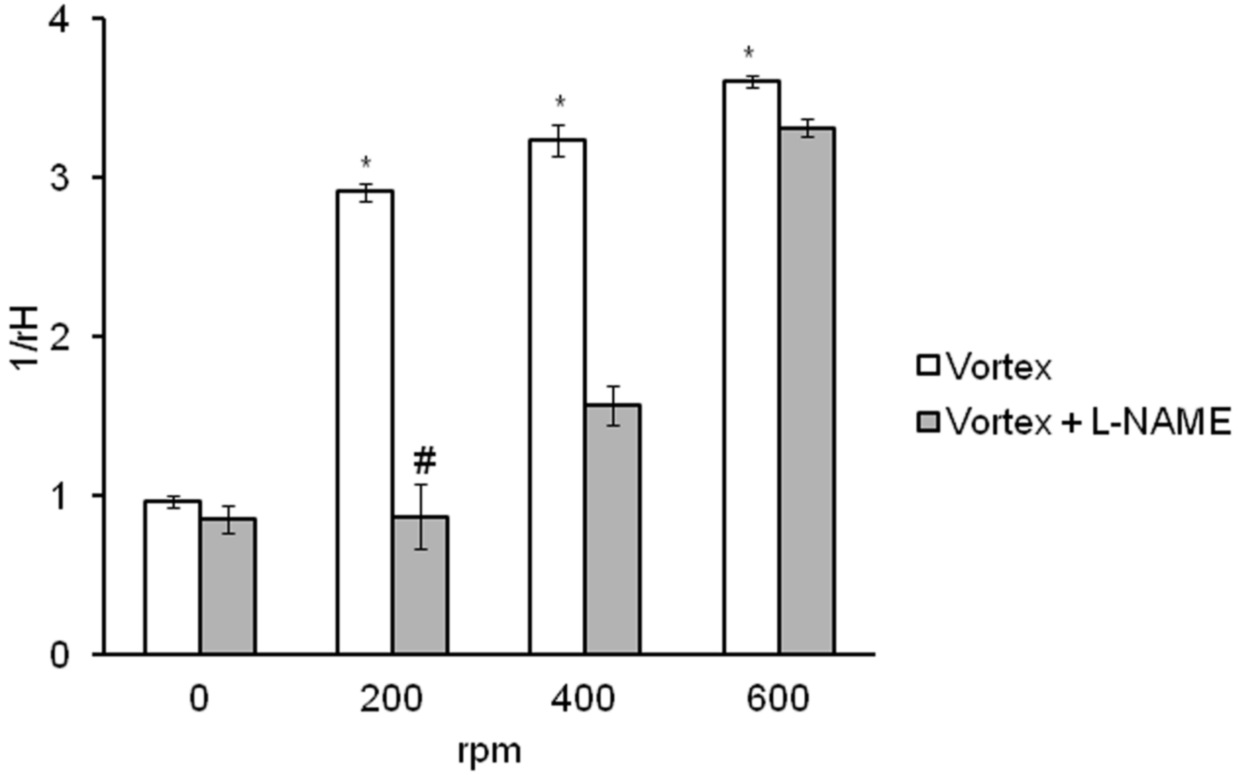

Supplementary Figure 2. Fluorescence anisotropy to measure fluidity of RBC membrane. Graph shows Anisotropy values which are inversely related to membrane fluidity. We observed significant increase in RBC membrane fluidity with increasing speeds of vortex (200, 400, and 600 rpm versus 0 rpm) (n = 3; *p < 0.004). The eNOS inhibitor (L-NAME) significantly decreased fluidity of membranes of vortexed RBC at 200 rpm (n = 3; #p < 0.001).

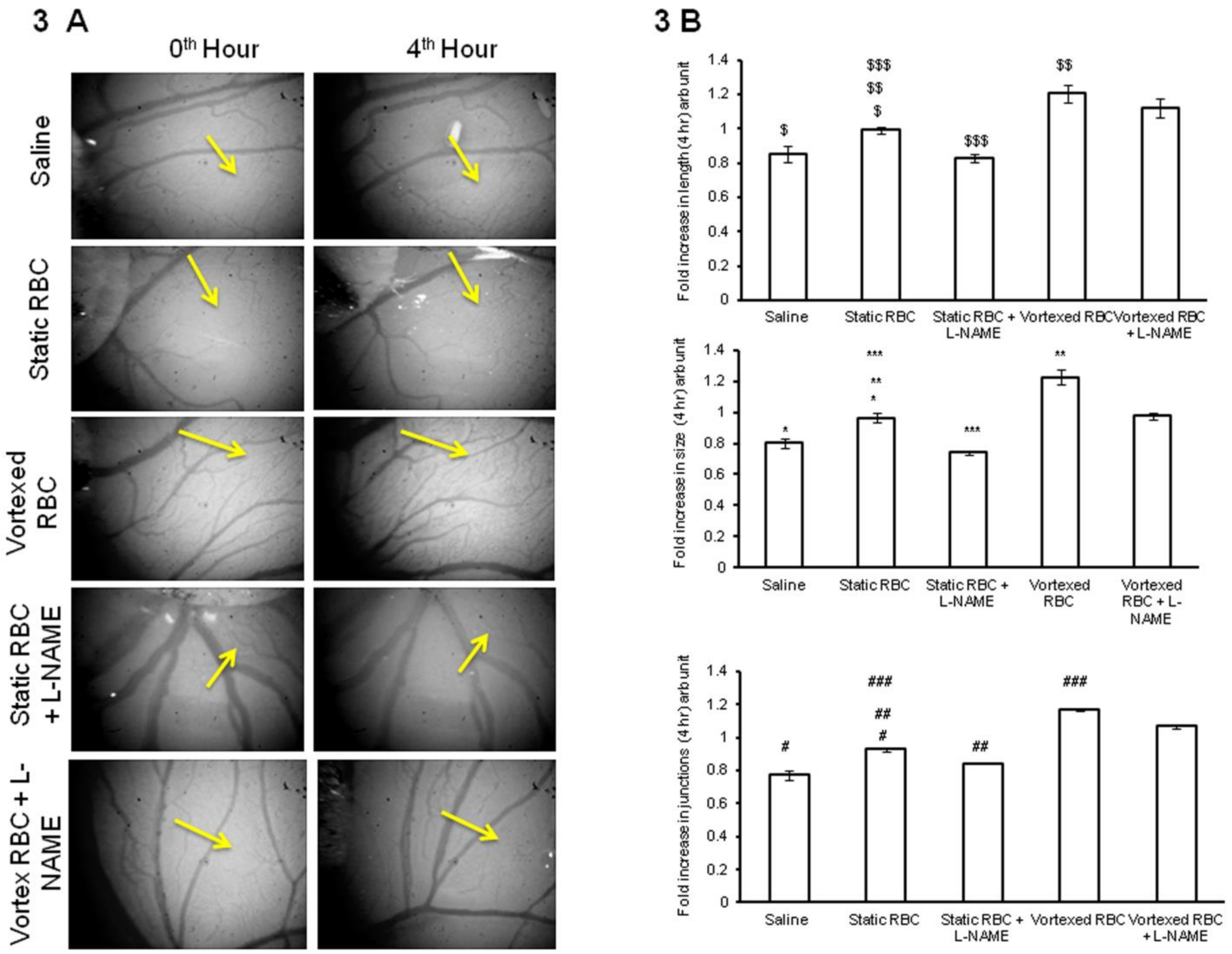

Supplementary Figure 3. Angiogenic effect of NO produced by vortexed RBC. (A) Representative images of angiogenesis assay showing blood vessel formation in vascular beds after 4 days treatment with static RBC or vortexed RBC. The images of the vessels adjacent to the RBC under this condition static, vortexed, static+L-NAME, vortexed+L-NAME treated RBC were taken with Nikon Cool Pix camera adapted to a stereo microscope. Images were converted to gray scale and presented. Yellow arrows showed the formation of new vessels in the area. **(B)** Analysis of the images was carried out using Angioquant software. Number of separate vessel size, length and junction complexes was analyzed by the software for 4th hour time point. Data presented as fold increase with time. Bar graph shows formation of vessels treated with static or vortexed RBC versus saline controls. Vessels treated with static RBC showed significant

reduction in size (n = 3; *p = 0.017; **p = 0.006), length (n = 3; \$$p$ = .034; \$\$$p$ = .011), and junction formation (n = 3; #p = 0.013; ##p = 0.007) compared with vessels treated with vortexed RBC. The eNOS dependence of RBC-NO production was confirmed by measurement of NO produced in static and vortexed RBC treated with or without L-NAME.

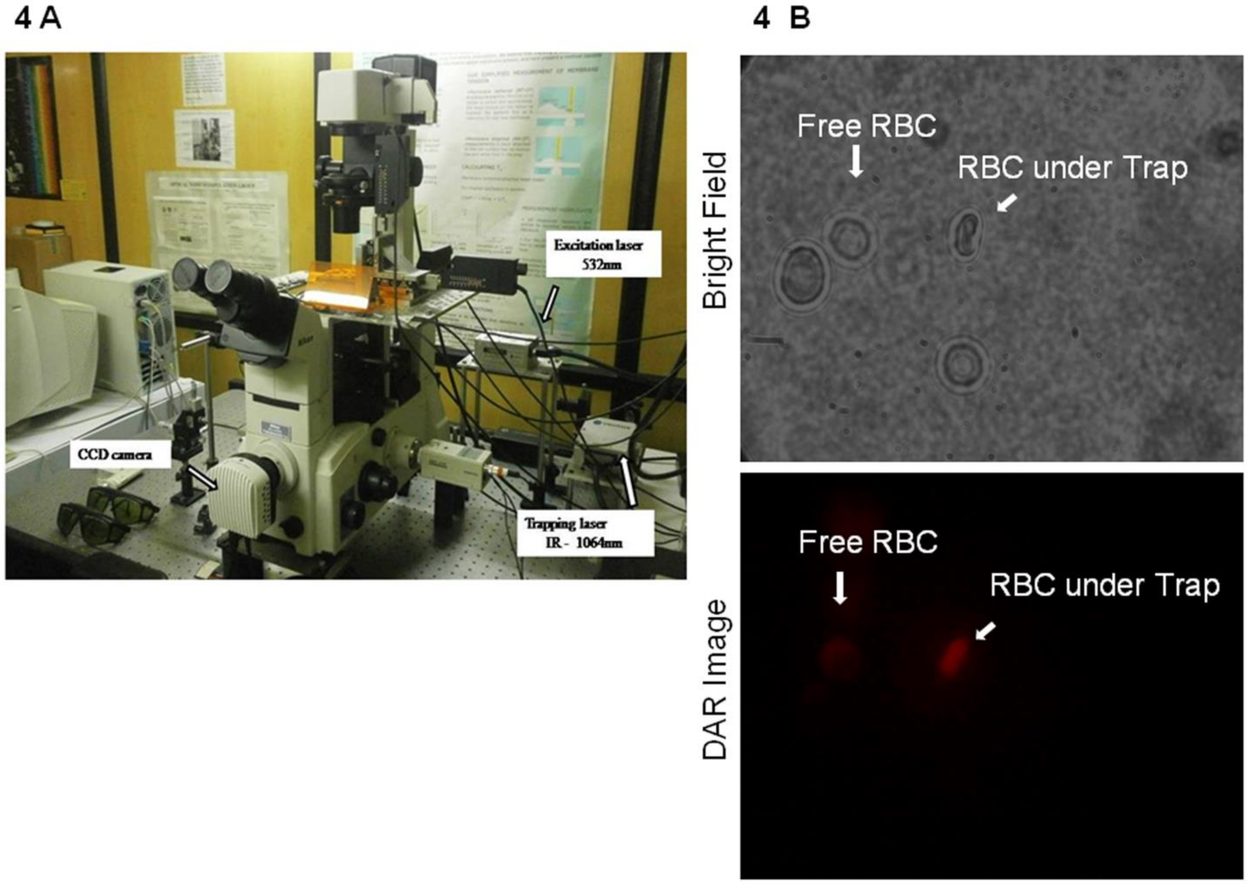

Supplementary Figure 4. Direct deformation by trapping RBC induces NO production. Individual human RBCs were deformed directly using optical traps with 0.004-0.05 pN/nm level of force. **(A)** Apparatus of optical tweezers and the optical tweezers set-up was built around an inverted microscope (TE-2000U, Nikon Corporation,Japan). A 1.5W 1064 nm Nd:YAG laser (Laser Quantum,UK) focused through an oil-immersion objective (Plan Apo, N.A. 1.4) was used to form an optical trap. **(B)** Image of DAR fluorescence showing NO production in trapped RBC versus a free RBC as a negative control.
